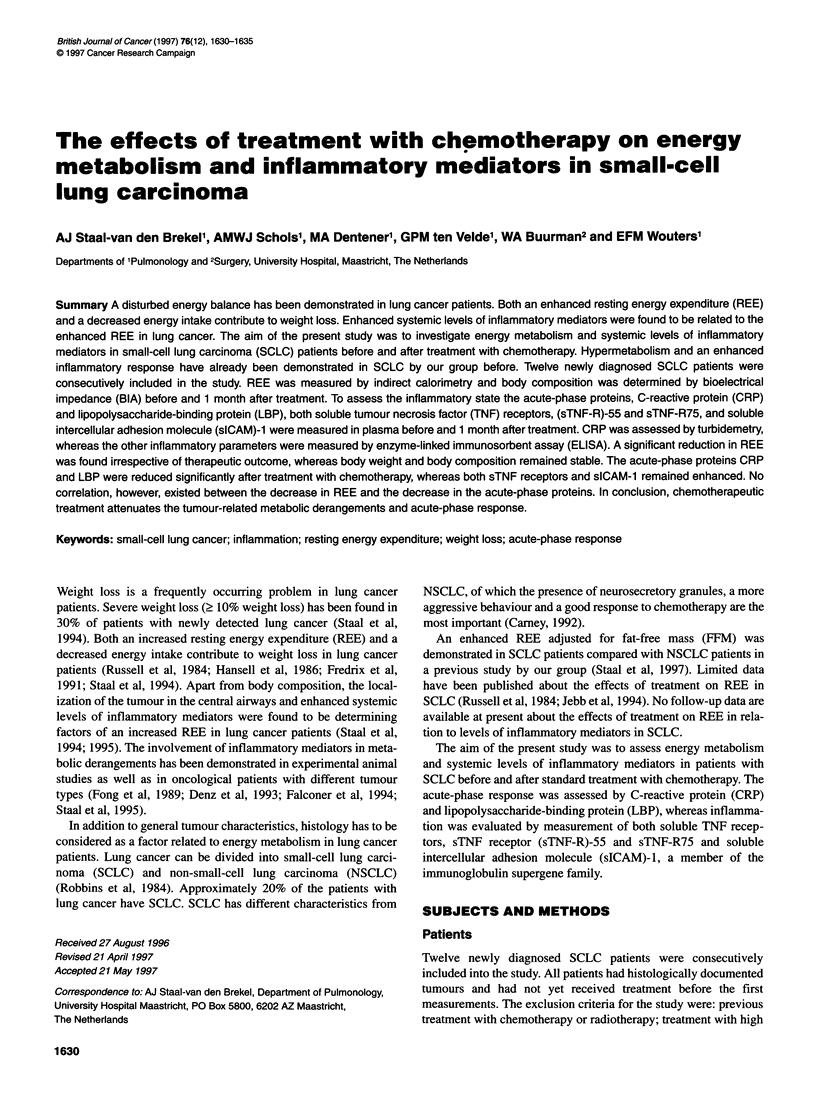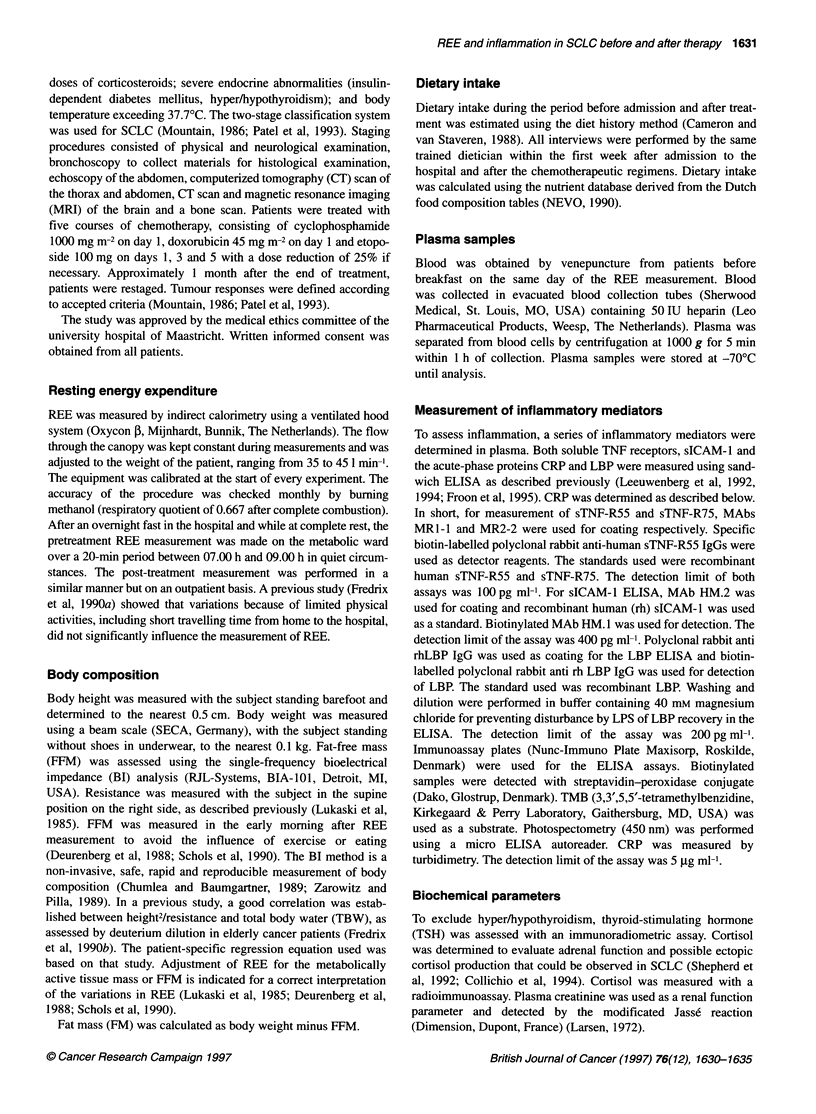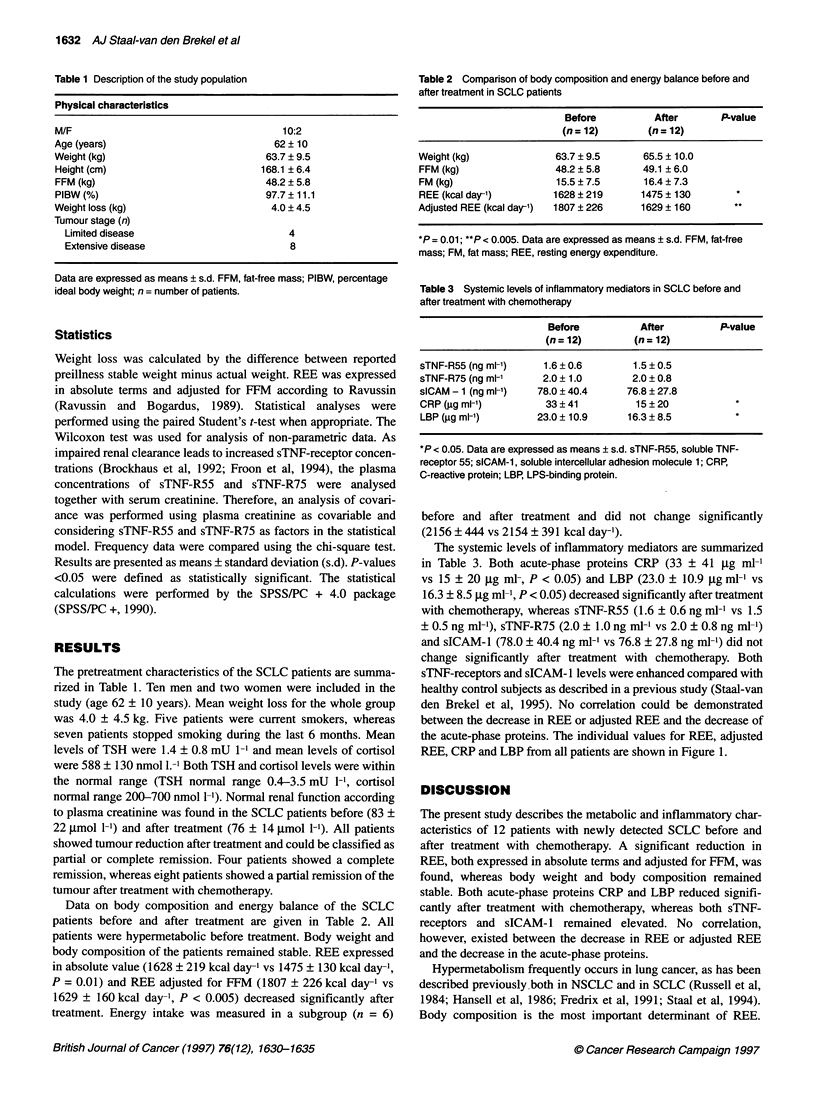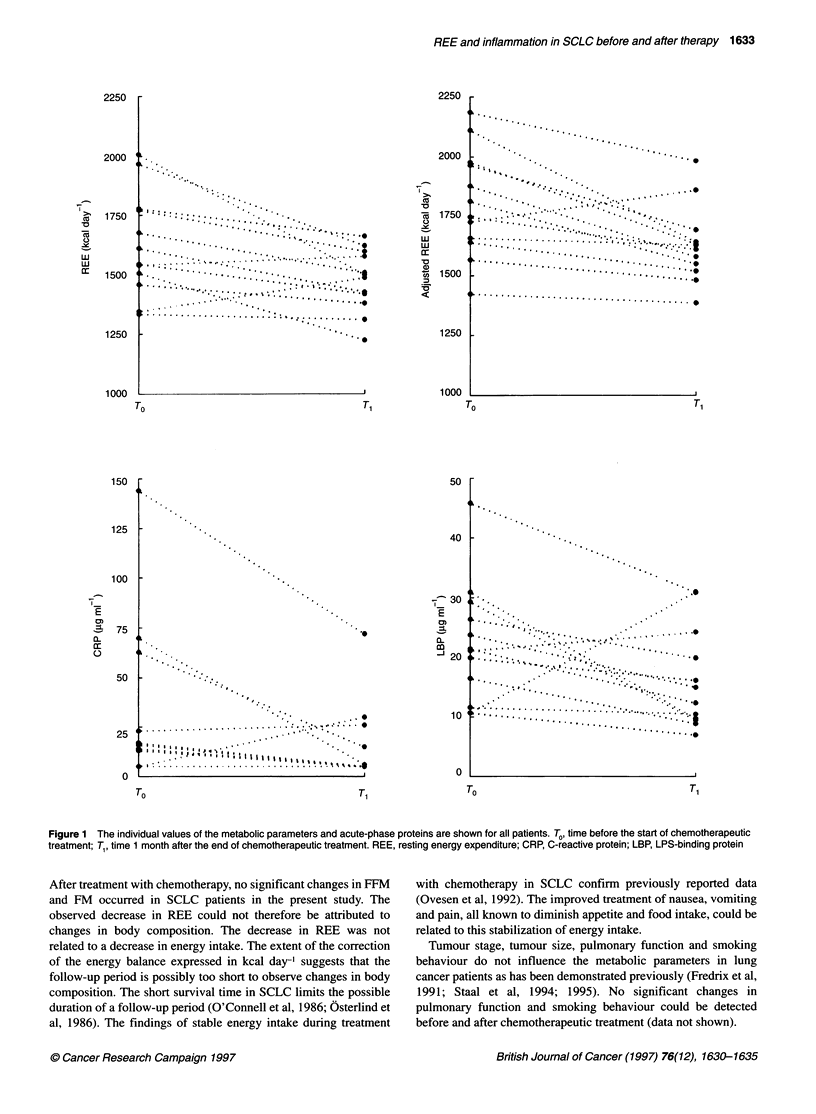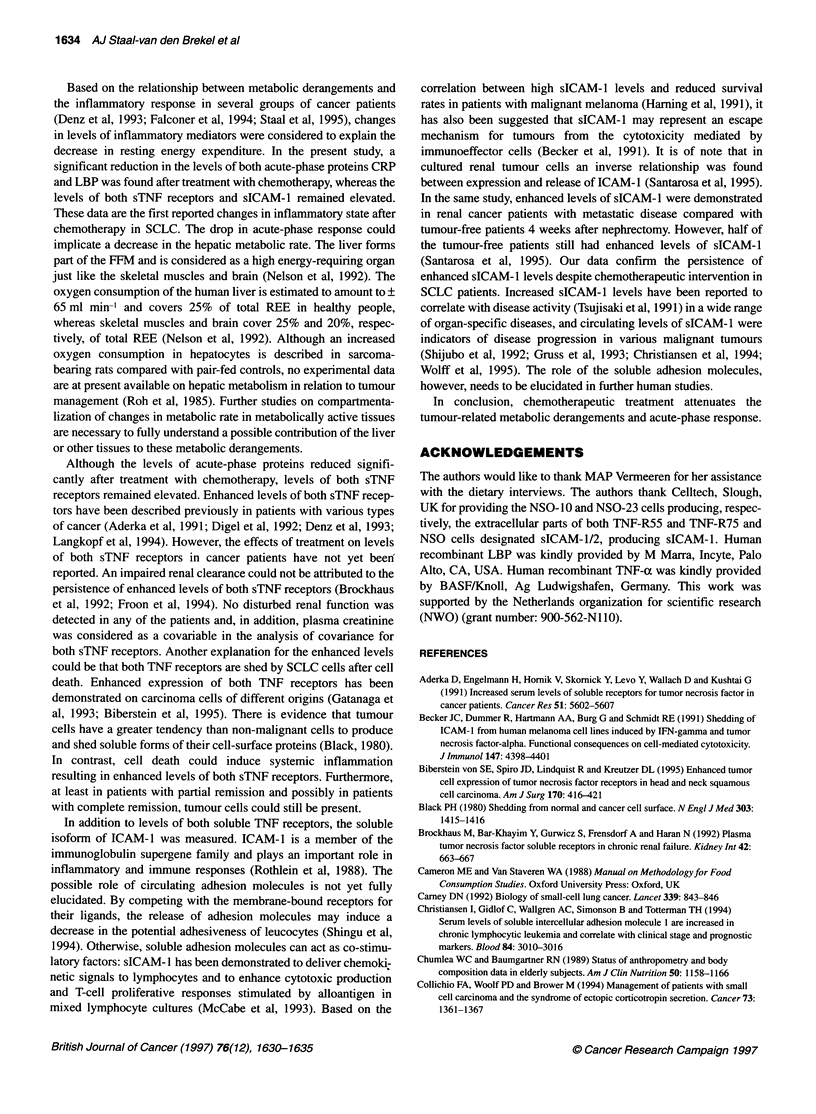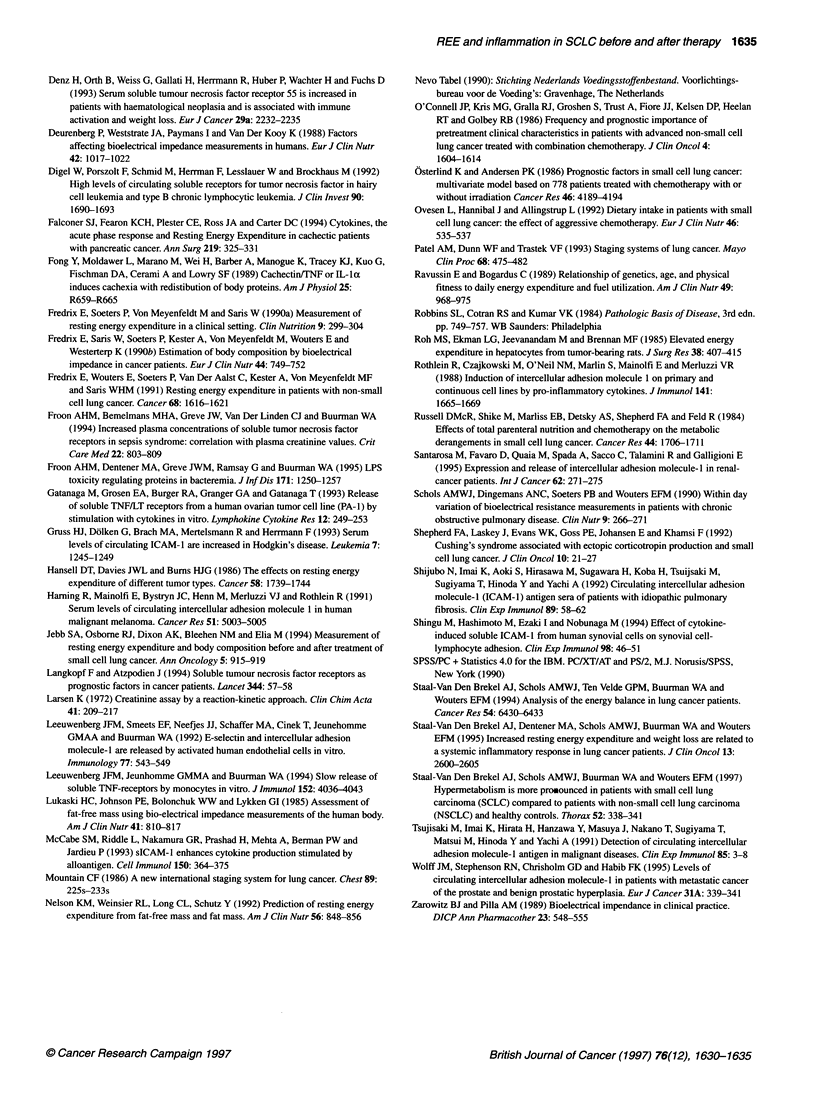# The effects of treatment with chemotherapy on energy metabolism and inflammatory mediators in small-cell lung carcinoma.

**DOI:** 10.1038/bjc.1997.608

**Published:** 1997

**Authors:** A. J. Staal-van den Brekel, A. M. Schols, M. A. Dentener, G. P. ten Velde, W. A. Buurman, E. F. Wouters

**Affiliations:** Department of Pulmonology, University Hospital, Maastricht, The Netherlands.

## Abstract

A disturbed energy balance has been demonstrated in lung cancer patients. Both an enhanced resting energy expenditure (REE) and a decreased energy intake contribute to weight loss. Enhanced systemic levels of inflammatory mediators were found to be related to the enhanced REE in lung cancer. The aim of the present study was to investigate energy metabolism and systemic levels of inflammatory mediators in small-cell lung carcinoma (SCLC) patients before and after treatment with chemotherapy. Hypermetabolism and an enhanced inflammatory response have already been demonstrated in SCLC by our group before. Twelve newly diagnosed SCLC patients were consecutively included in the study. REE was measured by indirect calorimetry and body composition was determined by bioelectrical impedance (BIA) before and 1 month after treatment. To assess the inflammatory state the acute-phase proteins, C-reactive protein (CRP) and lipopolysaccharide-binding protein (LBP), both soluble tumour necrosis factor (TNF) receptors, (sTNF-R)-55 and sTNF-R75, and soluble intercellular adhesion molecule (sICAM)-1 were measured in plasma before and 1 month after treatment. CRP was assessed by turbidemetry, whereas the other inflammatory parameters were measured by enzyme-linked immunosorbent assay (ELISA). A significant reduction in REE was found irrespective of therapeutic outcome, whereas body weight and body composition remained stable. The acute-phase proteins CRP and LBP were reduced significantly after treatment with chemotherapy, whereas both sTNF receptors and sICAM-1 remained enhanced. No correlation, however, existed between the decrease in REE and the decrease in the acute-phase proteins. In conclusion, chemotherapeutic treatment attenuates the tumour-related metabolic derangements and acute-phase response.


*British Journal of Cancer* (1997) **76**(12), 1630–1635
© 1997 Cancer Research Campaign

### The effects of treatment with chemotherapy on energy metabolism and inflammatory mediators in small-cell lung carcinoma

**AJ Staal-van den Brekel[1], AMWJ Schols[1], MA Dentener[1], GPM ten Velde[1], WA Buurman[2] and EFM Wouters[1]**

Departments of [1]Pulmonology and [2]Surgery, University Hospital, Maastricht, The Netherlands

**Summary** A disturbed energy balance has been demonstrated in lung cancer patients. Both an enhanced resting energy expenditure (REE) and a decreased energy intake contribute to weight loss. Enhanced systemic levels of inflammatory mediators were found to be related to the enhanced REE in lung cancer. The aim of the present study was to investigate energy metabolism and systemic levels of inflammatory mediators in small-cell lung carcinoma (SCLC) patients before and after treatment with chemotherapy. Hypermetabolism and an enhanced inflammatory response have already been demonstrated in SCLC by our group before. Twelve newly diagnosed SCLC patients were consecutively included in the study. REE was measured by indirect calorimetry and body composition was determined by bioelectrical impedance (BIA) before and 1 month after treatment. To assess the inflammatory state the acute-phase proteins, C-reactive protein (CRP) and lipopolysaccharide-binding protein (LBP), both soluble tumour necrosis factor (TNF) receptors, (sTNF-R)-55 and sTNF-R75, and soluble intercellular adhesion molecule (sICAM)-1 were measured in plasma before and 1 month after treatment. CRP was assessed by turbidemetry, whereas the other inflammatory parameters were measured by enzyme-linked immunosorbent assay (ELISA). A significant reduction in REE was found irrespective of therapeutic outcome, whereas body weight and body composition remained stable. The acute-phase proteins CRP and LBP were reduced significantly after treatment with chemotherapy, whereas both sTNF receptors and sICAM-1 remained enhanced. No correlation, however, existed between the decrease in REE and the decrease in the acute-phase proteins. In conclusion, chemotherapeutic treatment attenuates the tumour-related metabolic derangements and acute-phase response.

**Keywords:** small-cell lung cancer; inflammation; resting energy expenditure; weight loss; acute-phase response

Weight loss is a frequently occurring problem in lung cancer patients. Severe weight loss (≥ 10% weight loss) has been found in 30% of patients with newly detected lung cancer (Staal et al, 1994). Both an increased resting energy expenditure (REE) and a decreased energy intake contribute to weight loss in lung cancer patients (Russell et al, 1984; Hansell et al, 1986; Fredrix et al, 1991; Staal et al, 1994). Apart from body composition, the localization of the tumour in the central airways and enhanced systemic levels of inflammatory mediators were found to be determining factors of an increased REE in lung cancer patients (Staal et al, 1994; 1995). The involvement of inflammatory mediators in metabolic derangements has been demonstrated in experimental animal studies as well as in oncological patients with different tumour types (Fong et al, 1989; Denz et al, 1993; Falconer et al, 1994; Staal et al, 1995).

In addition to general tumour characteristics, histology has to be considered as a factor related to energy metabolism in lung cancer patients. Lung cancer can be divided into small-cell lung carcinoma (SCLC) and non-small-cell lung carcinoma (NSCLC) (Robbins et al, 1984). Approximately 20% of the patients with lung cancer have SCLC. SCLC has different characteristics from NSCLC, of which the presence of neurosecretory granules, a more aggressive behaviour and a good response to chemotherapy are the most important (Carney, 1992).

An enhanced REE adjusted for fat-free mass (FFM) was demonstrated in SCLC patients compared with NSCLC patients in a previous study by our group (Staal et al, 1997). Limited data have been published about the effects of treatment on REE in SCLC (Russell et al, 1984; Jebb et al, 1994). No follow-up data are available at present about the effects of treatment on REE in relation to levels of inflammatory mediators in SCLC.

The aim of the present study was to assess energy metabolism and systemic levels of inflammatory mediators in patients with SCLC before and after standard treatment with chemotherapy. The acute-phase response was assessed by C-reactive protein (CRP) and lipopolysaccharide-binding protein (LBP), whereas inflammation was evaluated by measurement of both soluble TNF receptors, sTNF receptor (sTNF-R)-55 and sTNF-R75 and soluble intercellular adhesion molecule (sICAM)-1, a member of the immunoglobulin supergene family.

*Received 27 August 1996*
*Revised 21 April 1997*
*Accepted 21 May 1997*

*Correspondence to:* AJ Staal-van den Brekel, Department of Pulmonology, University Hospital Maastricht, PO Box 5800, 6202 AZ Maastricht, The Netherlands

### SUBJECTS AND METHODS

### Patients

Twelve newly diagnosed SCLC patients were consecutively included into the study. All patients had histologically documented tumours and had not yet received treatment before the first measurements. The exclusion criteria for the study were: previous treatment with chemotherapy or radiotherapy; treatment with high doses of corticosteroids; severe endocrine abnormalities (insulin-dependent diabetes mellitus, hyper/hypothyroidism); and body temperature exceeding 37.7°C. The two-stage classification system was used for SCLC (Mountain, 1986; Patel et al, 1993). Staging procedures consisted of physical and neurological examination, bronchoscopy to collect materials for histological examination, echoscopy of the abdomen, computerized tomography (CT) scan of the thorax and abdomen, CT scan and magnetic resonance imaging (MRI) of the brain and a bone scan. Patients were treated with five courses of chemotherapy, consisting of cyclophosphamide 1000 mg $m^{-2}$ on day 1, doxorubicin 45 mg $m^{-2}$ on day 1 and etoposide 100 mg on days 1, 3 and 5 with a dose reduction of 25% if necessary. Approximately 1 month after the end of treatment, patients were restaged. Tumour responses were defined according to accepted criteria (Mountain, 1986; Patel et al, 1993).

The study was approved by the medical ethics committee of the university hospital of Maastricht. Written informed consent was obtained from all patients.

### Resting energy expenditure

REE was measured by indirect calorimetry using a ventilated hood system (Oxycon β, Mijnhardt, Bunnik, The Netherlands). The flow through the canopy was kept constant during measurements and was adjusted to the weight of the patient, ranging from 35 to 45 l $min^{-1}$. The equipment was calibrated at the start of every experiment. The accuracy of the procedure was checked monthly by burning methanol (respiratory quotient of 0.667 after complete combustion). After an overnight fast in the hospital and while at complete rest, the pretreatment REE measurement was made on the metabolic ward over a 20-min period between 07.00 h and 09.00 h in quiet circumstances. The post-treatment measurement was performed in a similar manner but on an outpatient basis. A previous study (Fredrix et al, 1990*a*) showed that variations because of limited physical activities, including short travelling time from home to the hospital, did not significantly influence the measurement of REE.

### Body composition

Body height was measured with the subject standing barefoot and determined to the nearest 0.5 cm. Body weight was measured using a beam scale (SECA, Germany), with the subject standing without shoes in underwear, to the nearest 0.1 kg. Fat-free mass (FFM) was assessed using the single-frequency bioelectrical impedance (BI) analysis (RJL-Systems, BIA-101, Detroit, MI, USA). Resistance was measured with the subject in the supine position on the right side, as described previously (Lukaski et al, 1985). FFM was measured in the early morning after REE measurement to avoid the influence of exercise or eating (Deurenberg et al, 1988; Schols et al, 1990). The BI method is a non-invasive, safe, rapid and reproducible measurement of body composition (Chumlea and Baumgartner, 1989; Zarowitz and Pilla, 1989). In a previous study, a good correlation was established between $height^2$/resistance and total body water (TBW), as assessed by deuterium dilution in elderly cancer patients (Fredrix et al, 1990*b*). The patient-specific regression equation used was based on that study. Adjustment of REE for the metabolically active tissue mass or FFM is indicated for a correct interpretation of the variations in REE (Lukaski et al, 1985; Deurenberg et al, 1988; Schols et al, 1990).

Fat mass (FM) was calculated as body weight minus FFM.

### Dietary intake

Dietary intake during the period before admission and after treatment was estimated using the diet history method (Cameron and van Staveren, 1988). All interviews were performed by the same trained dietician within the first week after admission to the hospital and after the chemotherapeutic regimens. Dietary intake was calculated using the nutrient database derived from the Dutch food composition tables (NEVO, 1990).

### Plasma samples

Blood was obtained by venepuncture from patients before breakfast on the same day of the REE measurement. Blood was collected in evacuated blood collection tubes (Sherwood Medical, St. Louis, MO, USA) containing 50 IU heparin (Leo Pharmaceutical Products, Weesp, The Netherlands). Plasma was separated from blood cells by centrifugation at 1000 ***g*** for 5 min within 1 h of collection. Plasma samples were stored at –70°C until analysis.

### Measurement of inflammatory mediators

To assess inflammation, a series of inflammatory mediators were determined in plasma. Both soluble TNF receptors, sICAM-1 and the acute-phase proteins CRP and LBP were measured using sandwich ELISA as described previously (Leeuwenberg et al, 1992, 1994; Froon et al, 1995). CRP was determined as described below. In short, for measurement of sTNF-R55 and sTNF-R75, MAbs MR1-1 and MR2-2 were used for coating respectively. Specific biotin-labelled polyclonal rabbit anti-human sTNF-R55 IgGs were used as detector reagents. The standards used were recombinant human sTNF-R55 and sTNF-R75. The detection limit of both assays was 100 pg $ml^{-1}$. For sICAM-1 ELISA, MAb HM.2 was used for coating and recombinant human (rh) sICAM-1 was used as a standard. Biotinylated MAb HM.1 was used for detection. The detection limit of the assay was 400 pg $ml^{-1}$. Polyclonal rabbit anti rhLBP IgG was used as coating for the LBP ELISA and biotin-labelled polyclonal rabbit anti rh LBP IgG was used for detection of LBP. The standard used was recombinant LBP. Washing and dilution were performed in buffer containing 40 mM magnesium chloride for preventing disturbance by LPS of LBP recovery in the ELISA. The detection limit of the assay was 200 pg $ml^{-1}$. Immunoassay plates (Nunc-Immuno Plate Maxisorp, Roskilde, Denmark) were used for the ELISA assays. Biotinylated samples were detected with streptavidin–peroxidase conjugate (Dako, Glostrup, Denmark). TMB (3,3′,5,5′-tetramethylbenzidine, Kirkegaard & Perry Laboratory, Gaithersburg, MD, USA) was used as a substrate. Photospectometry (450 nm) was performed using a micro ELISA autoreader. CRP was measured by turbidimetry. The detection limit of the assay was 5 μg $ml^{-1}$.

### Biochemical parameters

To exclude hyper/hypothyroidism, thyroid-stimulating hormone (TSH) was assessed with an immunoradiometric assay. Cortisol was determined to evaluate adrenal function and possible ectopic cortisol production that could be observed in SCLC (Shepherd et al, 1992; Collichio et al, 1994). Cortisol was measured with a radioimmunoassay. Plasma creatinine was used as a renal function parameter and detected by the modificated Jassé reaction (Dimension, Dupont, France) (Larsen, 1972).

© Cancer Research Campaign 1997

Table 1 Description of the study population

| Physical characteristics | |
|---|---|
| M/F | 10:2 |
| Age (years) | 62 ± 10 |
| Weight (kg) | 63.7 ± 9.5 |
| Height (cm) | 168.1 ± 6.4 |
| FFM (kg) | 48.2 ± 5.8 |
| PIBW (%) | 97.7 ± 11.1 |
| Weight loss (kg) | 4.0 ± 4.5 |
| Tumour stage (*n*) | |
| Limited disease | 4 |
| Extensive disease | 8 |

Data are expressed as means ± s.d. FFM, fat-free mass; PIBW, percentage ideal body weight; *n* = number of patients.

### Statistics

Weight loss was calculated by the difference between reported preillness stable weight minus actual weight. REE was expressed in absolute terms and adjusted for FFM according to Ravussin (Ravussin and Bogardus, 1989). Statistical analyses were performed using the paired Student's *t*-test when appropriate. The Wilcoxon test was used for analysis of non-parametric data. As impaired renal clearance leads to increased sTNF-receptor concentrations (Brockhaus et al, 1992; Froon et al, 1994), the plasma concentrations of sTNF-R55 and sTNF-R75 were analysed together with serum creatinine. Therefore, an analysis of covariance was performed using plasma creatinine as covariable and considering sTNF-R55 and sTNF-R75 as factors in the statistical model. Frequency data were compared using the chi-square test. Results are presented as means ± standard deviation (s.d). *P*-values <0.05 were defined as statistically significant. The statistical calculations were performed by the SPSS/PC + 4.0 package (SPSS/PC +, 1990).

### RESULTS

The pretreatment characteristics of the SCLC patients are summarized in Table 1. Ten men and two women were included in the study (age 62 ± 10 years). Mean weight loss for the whole group was 4.0 ± 4.5 kg. Five patients were current smokers, whereas seven patients stopped smoking during the last 6 months. Mean levels of TSH were 1.4 ± 0.8 mU $l^{-1}$ and mean levels of cortisol were 588 ± 130 nmol $l.^{-1}$ Both TSH and cortisol levels were within the normal range (TSH normal range 0.4–3.5 mU $l^{-1}$, cortisol normal range 200–700 nmol $l^{-1}$). Normal renal function according to plasma creatinine was found in the SCLC patients before (83 ± 22 μmol $l^{-1}$) and after treatment (76 ± 14 μmol $l^{-1}$). All patients showed tumour reduction after treatment and could be classified as partial or complete remission. Four patients showed a complete remission, whereas eight patients showed a partial remission of the tumour after treatment with chemotherapy.

Data on body composition and energy balance of the SCLC patients before and after treatment are given in Table 2. All patients were hypermetabolic before treatment. Body weight and body composition of the patients remained stable. REE expressed in absolute value (1628 ± 219 kcal $day^{-1}$ vs 1475 ± 130 kcal $day^{-1}$, $P = 0.01$) and REE adjusted for FFM (1807 ± 226 kcal $day^{-1}$ vs 1629 ± 160 kcal $day^{-1}$, $P < 0.005$) decreased significantly after treatment. Energy intake was measured in a subgroup ($n = 6$) before and after treatment and did not change significantly (2156 ± 444 vs 2154 ± 391 kcal $day^{-1}$).

Table 2 Comparison of body composition and energy balance before and after treatment in SCLC patients

| | Before (*n* = 12) | After (*n* = 12) | *P*-value |
|---|---|---|---|
| Weight (kg) | 63.7 ± 9.5 | 65.5 ± 10.0 | |
| FFM (kg) | 48.2 ± 5.8 | 49.1 ± 6.0 | |
| FM (kg) | 15.5 ± 7.5 | 16.4 ± 7.3 | |
| REE (kcal $day^{-1}$) | 1628 ± 219 | 1475 ± 130 | * |
| Adjusted REE (kcal $day^{-1}$) | 1807 ± 226 | 1629 ± 160 | ** |

**P* = 0.01; ***P* < 0.005. Data are expressed as means ± s.d. FFM, fat-free mass; FM, fat mass; REE, resting energy expenditure.

Table 3 Systemic levels of inflammatory mediators in SCLC before and after treatment with chemotherapy

| | Before (*n* = 12) | After (*n* = 12) | *P*-value |
|---|---|---|---|
| sTNF-R55 (ng $ml^{-1}$) | 1.6 ± 0.6 | 1.5 ± 0.5 | |
| sTNF-R75 (ng $ml^{-1}$ | 2.0 ± 1.0 | 2.0 ± 0.8 | |
| sICAM – 1 (ng $ml^{-1}$) | 78.0 ± 40.4 | 76.8 ± 27.8 | |
| CRP (μg $ml^{-1}$) | 33 ± 41 | 15 ± 20 | * |
| LBP (μg $ml^{-1}$) | 23.0 ± 10.9 | 16.3 ± 8.5 | * |

**P* < 0.05. Data are expressed as means ± s.d. sTNF-R55, soluble TNF-receptor 55; sICAM-1, soluble intercellular adhesion molecule 1; CRP, C-reactive protein; LBP, LPS-binding protein.

The systemic levels of inflammatory mediators are summarized in Table 3. Both acute-phase proteins CRP (33 ± 41 μg $ml^{-1}$ vs 15 ± 20 μg $ml^{-}$, $P < 0.05$) and LBP (23.0 ± 10.9 μg $ml^{-1}$ vs 16.3 ± 8.5 μg $ml^{-1}$, $P < 0.05$) decreased significantly after treatment with chemotherapy, whereas sTNF-R55 (1.6 ± 0.6 ng $ml^{-1}$ vs 1.5 ± 0.5 ng $ml^{-1}$), sTNF-R75 (2.0 ± 1.0 ng $ml^{-1}$ vs 2.0 ± 0.8 ng $ml^{-1}$) and sICAM-1 (78.0 ± 40.4 ng $ml^{-1}$ vs 76.8 ± 27.8 ng $ml^{-1}$) did not change significantly after treatment with chemotherapy. Both sTNF-receptors and sICAM-1 levels were enhanced compared with healthy control subjects as described in a previous study (Staal-van den Brekel et al, 1995). No correlation could be demonstrated between the decrease in REE or adjusted REE and the decrease of the acute-phase proteins. The individual values for REE, adjusted REE, CRP and LBP from all patients are shown in Figure 1.

### DISCUSSION

The present study describes the metabolic and inflammatory characteristics of 12 patients with newly detected SCLC before and after treatment with chemotherapy. A significant reduction in REE, both expressed in absolute terms and adjusted for FFM, was found, whereas body weight and body composition remained stable. Both acute-phase proteins CRP and LBP reduced significantly after treatment with chemotherapy, whereas both sTNF-receptors and sICAM-1 remained elevated. No correlation, however, existed between the decrease in REE or adjusted REE and the decrease in the acute-phase proteins.

Hypermetabolism frequently occurs in lung cancer, as has been described previously both in NSCLC and in SCLC (Russell et al, 1984; Hansell et al, 1986; Fredrix et al, 1991; Staal et al, 1994). Body composition is the most important determinant of REE.

© Cancer Research Campaign 1997

**Figure 1** The individual values of the metabolic parameters and acute-phase proteins are shown for all patients. $T_0$, time before the start of chemotherapeutic treatment; $T_1$, time 1 month after the end of chemotherapeutic treatment. REE, resting energy expenditure; CRP, C-reactive protein; LBP, LPS-binding protein

After treatment with chemotherapy, no significant changes in FFM and FM occurred in SCLC patients in the present study. The observed decrease in REE could not therefore be attributed to changes in body composition. The decrease in REE was not related to a decrease in energy intake. The extent of the correction of the energy balance expressed in kcal day$^{-1}$ suggests that the follow-up period is possibly too short to observe changes in body composition. The short survival time in SCLC limits the possible duration of a follow-up period (O'Connell et al, 1986; Österlind et al, 1986). The findings of stable energy intake during treatment with chemotherapy in SCLC confirm previously reported data (Ovesen et al, 1992). The improved treatment of nausea, vomiting and pain, all known to diminish appetite and food intake, could be related to this stabilization of energy intake.

Tumour stage, tumour size, pulmonary function and smoking behaviour do not influence the metabolic parameters in lung cancer patients as has been demonstrated previously (Fredrix et al, 1991; Staal et al, 1994; 1995). No significant changes in pulmonary function and smoking behaviour could be detected before and after chemotherapeutic treatment (data not shown).

© Cancer Research Campaign 1997 

Based on the relationship between metabolic derangements and the inflammatory response in several groups of cancer patients (Denz et al, 1993; Falconer et al, 1994; Staal et al, 1995), changes in levels of inflammatory mediators were considered to explain the decrease in resting energy expenditure. In the present study, a significant reduction in the levels of both acute-phase proteins CRP and LBP was found after treatment with chemotherapy, whereas the levels of both sTNF receptors and sICAM-1 remained elevated. These data are the first reported changes in inflammatory state after chemotherapy in SCLC. The drop in acute-phase response could implicate a decrease in the hepatic metabolic rate. The liver forms part of the FFM and is considered as a high energy-requiring organ just like the skeletal muscles and brain (Nelson et al, 1992). The oxygen consumption of the human liver is estimated to amount to ± 65 ml $min^{-1}$ and covers 25% of total REE in healthy people, whereas skeletal muscles and brain cover 25% and 20%, respectively, of total REE (Nelson et al, 1992). Although an increased oxygen consumption in hepatocytes is described in sarcoma-bearing rats compared with pair-fed controls, no experimental data are at present available on hepatic metabolism in relation to tumour management (Roh et al, 1985). Further studies on compartmentalization of changes in metabolic rate in metabolically active tissues are necessary to fully understand a possible contribution of the liver or other tissues to these metabolic derangements.

Although the levels of acute-phase proteins reduced significantly after treatment with chemotherapy, levels of both sTNF receptors remained elevated. Enhanced levels of both sTNF receptors have been described previously in patients with various types of cancer (Aderka et al, 1991; Digel et al, 1992; Denz et al, 1993; Langkopf et al, 1994). However, the effects of treatment on levels of both sTNF receptors in cancer patients have not yet been reported. An impaired renal clearance could not be attributed to the persistence of enhanced levels of both sTNF receptors (Brockhaus et al, 1992; Froon et al, 1994). No disturbed renal function was detected in any of the patients and, in addition, plasma creatinine was considered as a covariable in the analysis of covariance for both sTNF receptors. Another explanation for the enhanced levels could be that both TNF receptors are shed by SCLC cells after cell death. Enhanced expression of both TNF receptors has been demonstrated on carcinoma cells of different origins (Gatanaga et al, 1993; Biberstein et al, 1995). There is evidence that tumour cells have a greater tendency than non-malignant cells to produce and shed soluble forms of their cell-surface proteins (Black, 1980). In contrast, cell death could induce systemic inflammation resulting in enhanced levels of both sTNF receptors. Furthermore, at least in patients with partial remission and possibly in patients with complete remission, tumour cells could still be present.

In addition to levels of both soluble TNF receptors, the soluble isoform of ICAM-1 was measured. ICAM-1 is a member of the immunoglobulin supergene family and plays an important role in inflammatory and immune responses (Rothlein et al, 1988). The possible role of circulating adhesion molecules is not yet fully elucidated. By competing with the membrane-bound receptors for their ligands, the release of adhesion molecules may induce a decrease in the potential adhesiveness of leucocytes (Shingu et al, 1994). Otherwise, soluble adhesion molecules can act as co-stimulatory factors: sICAM-1 has been demonstrated to deliver chemokinetic signals to lymphocytes and to enhance cytotoxic production and T-cell proliferative responses stimulated by alloantigen in mixed lymphocyte cultures (McCabe et al, 1993). Based on the correlation between high sICAM-1 levels and reduced survival rates in patients with malignant melanoma (Harning et al, 1991), it has also been suggested that sICAM-1 may represent an escape mechanism for tumours from the cytotoxicity mediated by immunoeffector cells (Becker et al, 1991). It is of note that in cultured renal tumour cells an inverse relationship was found between expression and release of ICAM-1 (Santarosa et al, 1995). In the same study, enhanced levels of sICAM-1 were demonstrated in renal cancer patients with metastatic disease compared with tumour-free patients 4 weeks after nephrectomy. However, half of the tumour-free patients still had enhanced levels of sICAM-1 (Santarosa et al, 1995). Our data confirm the persistence of enhanced sICAM-1 levels despite chemotherapeutic intervention in SCLC patients. Increased sICAM-1 levels have been reported to correlate with disease activity (Tsujisaki et al, 1991) in a wide range of organ-specific diseases, and circulating levels of sICAM-1 were indicators of disease progression in various malignant tumours (Shijubo et al, 1992; Gruss et al, 1993; Christiansen et al, 1994; Wolff et al, 1995). The role of the soluble adhesion molecules, however, needs to be elucidated in further human studies.

In conclusion, chemotherapeutic treatment attenuates the tumour-related metabolic derangements and acute-phase response.

### ACKNOWLEDGEMENTS

The authors would like to thank MAP Vermeeren for her assistance with the dietary interviews. The authors thank Celltech, Slough, UK for providing the NSO-10 and NSO-23 cells producing, respectively, the extracellular parts of both TNF-R55 and TNF-R75 and NSO cells designated sICAM-1/2, producing sICAM-1. Human recombinant LBP was kindly provided by M Marra, Incyte, Palo Alto, CA, USA. Human recombinant TNF-α was kindly provided by BASF/Knoll, Ag Ludwigshafen, Germany. This work was supported by the Netherlands organization for scientific research (NWO) (grant number: 900-562-N110).

### REFERENCES

Aderka D, Engelmann H, Hornik V, Skornick Y, Levo Y, Wallach D and Kushtai G (1991) Increased serum levels of soluble receptors for tumor necrosis factor in cancer patients. *Cancer Res* **51**: 5602–5607

Becker JC, Dummer R, Hartmann AA, Burg G and Schmidt RE (1991) Shedding of ICAM-1 from human melanoma cell lines induced by IFN-gamma and tumor necrosis factor-alpha. Functional consequences on cell-mediated cytotoxicity. *J Immunol* **147**: 4398–4401

Biberstein von SE, Spiro JD, Lindquist R and Kreutzer DL (1995) Enhanced tumor cell expression of tumor necrosis factor receptors in head and neck squamous cell carcinoma. *Am J Surg* **170**: 416–421

Black PH (1980) Shedding from normal and cancer cell surface. *N Engl J Med* **303**: 1415–1416

Brockhaus M, Bar-Khayim Y, Gurwicz S, Frensdorf A and Haran N (1992) Plasma tumor necrosis factor soluble receptors in chronic renal failure. *Kidney Int* **42**: 663–667

Cameron ME and Van Staveren WA (1988) *Manual on Methodology for Food Consumption Studies*. Oxford University Press: Oxford, UK

Carney DN (1992) Biology of small-cell lung cancer. *Lancet* **339**: 843–846

Christiansen I, Gidlof C, Wallgren AC, Simonson B and Totterman TH (1994) Serum levels of soluble intercellular adhesion molecule 1 are increased in chronic lymphocytic leukemia and correlate with clinical stage and prognostic markers. *Blood* **84**: 3010–3016

Chumlea WC and Baumgartner RN (1989) Status of anthropometry and body composition data in elderly subjects. *Am J Clin Nutrition* **50**: 1158–1166

Collichio FA, Woolf PD and Brower M (1994) Management of patients with small cell carcinoma and the syndrome of ectopic corticotropin secretion. *Cancer* **73**: 1361–1367

© Cancer Research Campaign 1997

Denz H, Orth B, Weiss G, Gallati H, Herrmann R, Huber P, Wachter H and Fuchs D (1993) Serum soluble tumour necrosis factor receptor 55 is increased in patients with haematological neoplasia and is associated with immune activation and weight loss. *Eur J Cancer* **29a**: 2232–2235

Deurenberg P, Weststrate JA, Paymans I and Van Der Kooy K (1988) Factors affecting bioelectrical impedance measurements in humans. *Eur J Clin Nutr* **42**: 1017–1022

Digel W, Porszolt F, Schmid M, Herrman F, Lesslauer W and Brockhaus M (1992) High levels of circulating soluble receptors for tumor necrosis factor in hairy cell leukemia and type B chronic lymphocytic leukemia. *J Clin Invest* **90**: 1690–1693

Falconer SJ, Fearon KCH, Plester CE, Ross JA and Carter DC (1994) Cytokines, the acute phase response and Resting Energy Expenditure in cachectic patients with pancreatic cancer. *Ann Surg* **219**: 325–331

Fong Y, Moldawer L, Marano M, Wei H, Barber A, Manogue K, Tracey KJ, Kuo G, Fischman DA, Cerami A and Lowry SF (1989) Cachectin/TNF or IL-1α induces cachexia with redistibution of body proteins. *Am J Physiol* **25**: R659–R665

Fredrix E, Soeters P, Von Meyenfeldt M and Saris W (1990a) Measurement of resting energy expenditure in a clinical setting. *Clin Nutrition* **9**: 299–304

Fredrix E, Saris W, Soeters P, Kester A, Von Meyenfeldt M, Wouters E and Westerterp K (1990*b*) Estimation of body composition by bioelectrical impedance in cancer patients. *Eur J Clin Nutr* **44**: 749–752

Fredrix E, Wouters E, Soeters P, Van Der Aalst C, Kester A, Von Meyenfeldt MF and Saris WHM (1991) Resting energy expenditure in patients with non-small cell lung cancer. *Cancer* **68**: 1616–1621

Froon AHM, Bemelmans MHA, Greve JW, Van Der Linden CJ and Buurman WA (1994) Increased plasma concentrations of soluble tumor necrosis factor receptors in sepsis syndrome: correlation with plasma creatinine values. *Crit Care Med* **22**: 803–809

Froon AHM, Dentener MA, Greve JWM, Ramsay G and Buurman WA (1995) LPS toxicity regulating proteins in bacteremia. *J Inf Dis* **171**: 1250–1257

Gatanaga M, Grosen EA, Burger RA, Granger GA and Gatanaga T (1993) Release of soluble TNF/LT receptors from a human ovarian tumor cell line (PA-1) by stimulation with cytokines in vitro. *Lymphokine Cytokine Res* **12**: 249–253

Gruss HJ, Dölken G, Brach MA, Mertelsmann R and Herrmann F (1993) Serum levels of circulating ICAM-1 are increased in Hodgkin's disease. *Leukemia* **7**: 1245–1249

Hansell DT, Davies JWL and Burns HJG (1986) The effects on resting energy expenditure of different tumor types. *Cancer* **58**: 1739–1744

Harning R, Mainolfi E, Bystryn JC, Henn M, Merluzzi VJ and Rothlein R (1991) Serum levels of circulating intercellular adhesion molecule 1 in human malignant melanoma. *Cancer Res* **51**: 5003–5005

Jebb SA, Osborne RJ, Dixon AK, Bleehen NM and Elia M (1994) Measurement of resting energy expenditure and body composition before and after treatment of small cell lung cancer. *Ann Oncology* **5**: 915–919

Langkopf F and Atzpodien J (1994) Soluble tumour necrosis factor receptors as prognostic factors in cancer patients. *Lancet* **344**: 57–58

Larsen K (1972) Creatinine assay by a reaction-kinetic approach. *Clin Chim Acta* **41**: 209–217

Leeuwenberg JFM, Smeets EF, Neefjes JJ, Schaffer MA, Cinek T, Jeunehomme GMAA and Buurman WA (1992) E-selectin and intercellular adhesion molecule-1 are released by activated human endothelial cells in vitro. *Immunology* **77**: 543–549

Leeuwenberg JFM, Jeunhomme GMMA and Buurman WA (1994) Slow release of soluble TNF-receptors by monocytes in vitro. *J Immunol* **152**: 4036–4043

Lukaski HC, Johnson PE, Bolonchuk WW and Lykken GI (1985) Assessment of fat-free mass using bio-electrical impedance measurements of the human body. *Am J Clin Nutr* **41**: 810–817

McCabe SM, Riddle L, Nakamura GR, Prashad H, Mehta A, Berman PW and Jardieu P (1993) sICAM-1 enhances cytokine production stimulated by alloantigen. *Cell Immunol* **150**: 364–375

Mountain CF (1986) A new international staging system for lung cancer. *Chest* **89**: 225s–233s

Nelson KM, Weinsier RL, Long CL, Schutz Y (1992) Prediction of resting energy expenditure from fat-free mass and fat mass. *Am J Clin Nutr* **56**: 848–856

Nevo Tabel (1990): *Stichting Nederlands Voedingsstoffenbestand.* Voorlichtingsbureau voor de Voeding's: Gravenhage, The Netherlands

O'Connell JP, Kris MG, Gralla RJ, Groshen S, Trust A, Fiore JJ, Kelsen DP, Heelan RT and Golbey RB (1986) Frequency and prognostic importance of pretreatment clinical characteristics in patients with advanced non-small cell lung cancer treated with combination chemotherapy. *J Clin Oncol* **4**: 1604–1614

Österlind K and Andersen PK (1986) Prognostic factors in small cell lung cancer: multivariate model based on 778 patients treated with chemotherapy with or without irradiation *Cancer Res* **46**: 4189–4194

Ovesen L, Hannibal J and Allingstrup L (1992) Dietary intake in patients with small cell lung cancer: the effect of aggressive chemotherapy. *Eur J Clin Nutr* **46**: 535–537

Patel AM, Dunn WF and Trastek VF (1993) Staging systems of lung cancer. *Mayo Clin Proc* **68**: 475–482

Ravussin E and Bogardus C (1989) Relationship of genetics, age, and physical fitness to daily energy expenditure and fuel utilization. *Am J Clin Nutr* **49**: 968–975

Robbins SL, Cotran RS and Kumar VK (1984) *Pathologic Basis of Disease*, 3rd edn. pp. 749–757. WB Saunders: Philadelphia

Roh MS, Ekman LG, Jeevanandam M and Brennan MF (1985) Elevated energy expenditure in hepatocytes from tumor-bearing rats. *J Surg Res* **38**: 407–415

Rothlein R, Czajkowski M, O'Neil NM, Marlin S, Mainolfi E and Merluzzi VR (1988) Induction of intercellular adhesion molecule 1 on primary and continuous cell lines by pro-inflammatory cytokines. *J Immunol* **141**: 1665–1669

Russell DMcR, Shike M, Marliss EB, Detsky AS, Shepherd FA and Feld R (1984) Effects of total parenteral nutrition and chemotherapy on the metabolic derangements in small cell lung cancer. *Cancer Res* **44**: 1706–1711

Santarosa M, Favaro D, Quaia M, Spada A, Sacco C, Talamini R and Galligioni E (1995) Expression and release of intercellular adhesion molecule-1 in renal-cancer patients. *Int J Cancer* **62**: 271–275

Schols AMWJ, Dingemans ANC, Soeters PB and Wouters EFM (1990) Within day variation of bioelectrical resistance measurements in patients with chronic obstructive pulmonary disease. *Clin Nutr* **9**: 266–271

Shepherd FA, Laskey J, Evans WK, Goss PE, Johansen E and Khamsi F (1992) Cushing's syndrome associated with ectopic corticotropin production and small cell lung cancer. *J Clin Oncol* **10**: 21–27

Shijubo N, Imai K, Aoki S, Hirasawa M, Sugawara H, Koba H, Tsuijsaki M, Sugiyama T, Hinoda Y and Yachi A (1992) Circulating intercellular adhesion molecule-1 (ICAM-1) antigen sera of patients with idiopathic pulmonary fibrosis. *Clin Exp Immunol* **89**: 58–62

Shingu M, Hashimoto M, Ezaki I and Nobunaga M (1994) Effect of cytokine-induced soluble ICAM-1 from human synovial cells on synovial cell-lymphocyte adhesion. *Clin Exp Immunol* **98**: 46–51

SPSS/PC + Statistics 4.0 for the IBM. PC/XT/AT and PS/2, M.J. Norusis/SPSS, New York (1990)

Staal-Van Den Brekel AJ, Schols AMWJ, Ten Velde GPM, Buurman WA and Wouters EFM (1994) Analysis of the energy balance in lung cancer patients. *Cancer Res* **54**: 6430–6433

Staal-Van Den Brekel AJ, Dentener MA, Schols AMWJ, Buurman WA and Wouters EFM (1995) Increased resting energy expenditure and weight loss are related to a systemic inflammatory response in lung cancer patients. *J Clin Oncol* **13**: 2600–2605

Staal-Van Den Brekel AJ, Schols AMWJ, Buurman WA and Wouters EFM (1997) Hypermetabolism is more pronounced in patients with small cell lung carcinoma (SCLC) compared to patients with non-small cell lung carcinoma (NSCLC) and healthy controls. *Thorax* **52**: 338–341

Tsujisaki M, Imai K, Hirata H, Hanzawa Y, Masuya J, Nakano T, Sugiyama T, Matsui M, Hinoda Y and Yachi A (1991) Detection of circulating intercellular adhesion molecule-1 antigen in malignant diseases. *Clin Exp Immunol* **85**: 3–8

Wolff JM, Stephenson RN, Chrisholm GD and Habib FK (1995) Levels of circulating intercellular adhesion molecule-1 in patients with metastatic cancer of the prostate and benign prostatic hyperplasia. *Eur J Cancer* **31A**: 339–341

Zarowitz BJ and Pilla AM (1989) Bioelectrical impendance in clinical practice. *DICP Ann Pharmacother* **23**: 548–555

© Cancer Research Campaign 1997